# Supplementary material for: Splice-Junction-Based Mapping of Alternative Isoforms in the Human Proteome
Source: Cell Rep. Author manuscript; Available in PMC 2020 Jan 15. (PMC6961840; doi:10.1016/j.celrep.2019.11.026)
Supplement: 2 [file NIHMS1546469-supplement-2.zip › DF1/PXD006675/SmoothMuscleCells/SmoothMuscleCells_9_NECAB3_EGLQSGPVCDALPAAG.pdf]

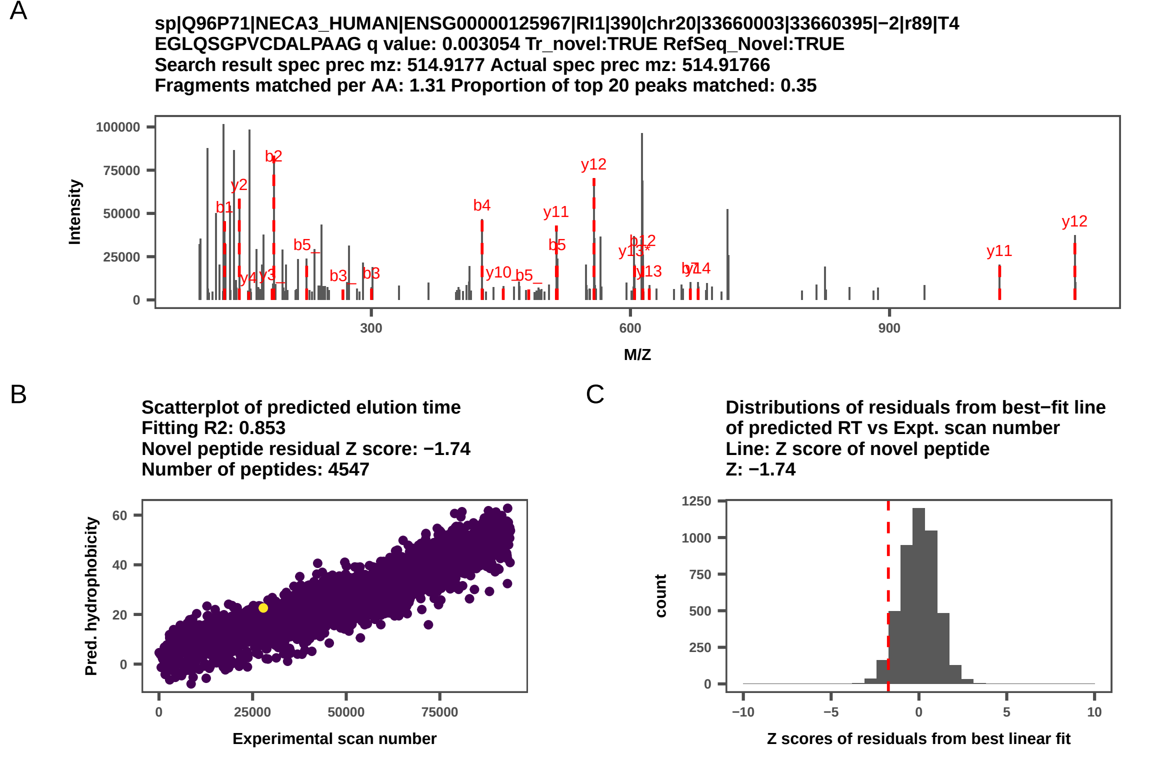
A
sp|Q96P71|NECA3_HUMAN|ENSG00000125967|RI1|390|chr20|33660003|33660395|−2|r89|T4
EGLQSGPVCDALPAAG q value: 0.003054 Tr_novel:TRUE RefSeq_Novel:TRUE
Search result spec prec mz: 514.9177 Actual spec prec mz: 514.91766
Fragments matched per AA: 1.31 Proportion of top 20 peaks matched: 0.35
Intensity
100000
75000
50000
25000
0
b1
y2
b2
b5_
y4
y3_
b3_
b3
b4
y10
b5_
y11
b5
y12
y13*
b12
y13
b14
y14
y11
y12
300
600
900
M/Z
B
Scatterplot of predicted elution time
Fitting R2: 0.853
Novel peptide residual Z score: −1.74
Number of peptides: 4547
Pred. hydrophobicity
60
40
20
0
0
25000
50000
75000
Experimental scan number
C
Distributions of residuals from best−fit line
of predicted RT vs Expt. scan number
Line: Z score of novel peptide
Z: −1.74
count
1250
1000
750
500
250
0
−10
−5
0
5
10
Z scores of residuals from best linear fit
